# Personality traits and end-of-life planning in older adults: insights from a population-based survey

**DOI:** 10.1007/s10433-026-00916-x

**Published:** 2026-03-13

**Authors:** Valérie-Anne Ryser, Sarah Vilpert, Jürgen Maurer

**Affiliations:** 1 Swiss Centre of Expertise in the Social Sciences (FORS), c/o University of Lausanne, Bâtiment Géopolis, 1015 Lausanne, Switzerland; 2 Faculty of Business and Economics, University of Lausanne, Lausanne, Switzerland

**Keywords:** Big Five, End-of-life, Nationwide study, Survey of Health Ageing and Retirement in Europe (SHARE), Switzerland

## Abstract

Personality traits (PTs) such as openness to experience, conscientiousness, extraversion, agreeableness, and neuroticism reflect individuals’ differences in thinking, feeling, and behaving. This study explores the association between these PTs and individuals’ commitment to end-of-life (EoL) planning. Understanding this association can provide valuable insights for professionals and family members, helping them effectively motivate individuals to engage in EoL planning, thereby contributing to an improved quality of life in the last stage. Using data from a self-completion questionnaire (n = 1,524) of SHARE Switzerland, this study employs logistic regression to explore the relationship between PTs measured by the Big Five Inventory Ten (BFI-10) and EoL planning attitudes and behaviours measured by well-established indicators: contemplating death, discussing one’s EoL preferences, organizing legal matters concerning EoL such as writing a testament and durable powers of attorney, designating a healthcare proxy, and completing a living will in case of potential future incapacity. Older adults with higher levels of openness think about and discuss EoL matters more frequently than individuals with lower levels of openness. They are also more likely to have a testament and a durable power of attorney. Older adults with higher levels of extraversion are more likely to have a testament, while participants scoring higher in neuroticism tend to ponder their EoL wishes more frequently. PTs’ contribution to participation in EoL planning appears to be relatively limited. Therefore, it is unlikely that PTs pose substantial impediments to engaging in comprehensive EoL planning, which can be promoted among individuals irrespective of their specific PTs.

## Introduction

End-of-life (EoL) planning refers to expressing one’s preferences for care and decisions at the end of life (Carr and Luth 2016, 2017). In practice, it involves reflecting on personal wishes, communicating them, and formalizing them through legal instruments, such as advance directives (Borrat-Besson et al. 2020; Vilpert et al. 2018). Such planning is linked to a better quality of life during this critical phase (Carr and Luth 2016, 2017). These benefits become particularly important  at older ages, given the health challenges in later life, like frailty (e.g. Mitnitski et al. 2015), chronic conditions, co-morbidity (e.g. Barnett et al. 2012; Marengoni et al. 2008; Melis et al. 2014; Onder et al. 2015), and cognitive impairment (e.g. Farina et al. 2022; Jansen et al. 2018). Therefore, it is widely recommended to plan in advance and proactively for the EoL to help those affected prepare for these challenges.

This study draws on a national random sample of the Swiss SHARE survey (Börsch-Supan et al. 2013), to explore how personality traits (PTs) like openness to experience, conscientiousness, extraversion, agreeableness, and neuroticism (John et al. 2008; John and Srivastava 1999; McCrae and Costa 2008) relate to EoL planning. Considerable attention has been devoted to the relationships between advance directives—that ensure that a person’s healthcare preferences are known and respected through legal documents, guiding family members and healthcare providers during critical times (Carr and Luth 2017)- and sociodemographic characteristics (e.g. Brinkman-Stoppelenburg et al. 2014; Carr 2012; Jimenez et al. 2018; Vilpert et al. 2018). However, other aspects of EoL planning have only received limited attention, and to our knowledge, little is known about the potential role of stable individual-level factors, such as PTs. Because EoL decisions are inherently shaped by how individuals think, feel, and behave, and given that PTs reflect these dimensions, examining PTs provides a crucial lens for understanding how such psychological dispositions may influence planning in this sensitive context. Understanding these links can help professionals and families support individuals more effectively.

This study addresses two gaps in the field: the first key strength is its comprehensive coverage of EoL planning components, enabling a holistic approach beyond the study of advance directives only (e.g. Brinkman-Stoppelenburg et al. 2014; Carr 2012; Jimenez et al. 2018; Vilpert et al. 2018), to include attitudes and behaviours related to medical and legal issues, dimensions that have received limited attention. Second, we investigate the association between PTs and these comprehensive EoL planning components.

### End-of-life planning

EoL planning is a highly personal and proactive process that empowers individuals to make anticipated choices aligned with their values, beliefs, and preferences (Carr 2003). This proactive approach for ageing individuals has been shown to significantly improve their well-being and quality of life for themselves and their families (Carr and Luth 2016, 2017). Conscientiously planning and regulating one’s EoL trajectory has yielded multifaceted benefits encompassing psychological, relational, and practical dimensions (Malhotra et al. 2022). It gives individuals greater control over the ultimate phase of their existence, fosters a sense of autonomy, reduces distress, and cultivates a greater understanding of peace and preparedness (Carr 2003; Detering et al. 2010). Moreover, structured EoL planning facilitates constructive family and professional discussions (Hong et al. 2016; Keeley 2017), diminishes the burden of decision-making on loved ones (Wendler and Rid 2011), and helps avert conflicts arising from unclear directives (Carr and Luth 2019). Overall, addressing death proactively empowers individuals and promotes a dignified EoL.

Advance care directives are the most documented EoL planning components (e.g. Brinkman-Stoppelenburg et al. 2014; Carr 2012; Jimenez et al. 2018); within the Swiss context, the relationship between advance directives and sociodemographic characteristics has been thoroughly investigated (Vilpert et al. 2018). However, to our knowledge, the broader aspects of EoL planning have been relatively underexplored and often studied in isolation (Carr 2012). In contrast, older adults tend to adopt a multidimensional approach to EoL considerations, extending beyond care-related concerns. This approach encompasses not only formal legal documents, such as advance directives, but also includes psychosocial, control, and burden-related aspects (Borrat-Besson et al. 2020, 2022). This process consists firstly of forming attitudes that facilitate and guide effective EoL planning and secondly of the actual planning behaviours that formalize and document an individual’s EoL decisions through, say, advance directives or the writing of a testament (Sudore et al. 2008). Attitudes include the extent to which individuals either engage in or avoid contemplating death, the frequency of pondering their EoL wishes, and their propensity for discussing important EoL issues with others. Behaviours concern the completion of a power of attorney, which authorizes a designated person to manage administrative matters on behalf of another person in defined circumstances, and the writing of a testament that designates the beneficiaries of a person’s assets and distribution. In the medical context, EoL care planning documents include living wills, which express a person’s medical treatment preferences and/or refusal in case of permanent or temporary loss of decision-making capacity, and the designation of a potential healthcare proxy authorized to make substitute medical decisions in incapacity.

This research, therefore, aims to explore these various dimensions of EoL planning holistically, including financial considerations (such as testament, and power of attorney), care decisions, and individuals’ attitudes towards anticipating the last stage of life. In addition, we examine the potential influence of stable individual factors, particularly PTs, on broader engagement, as EoL planning has not been sufficiently studied. This study aims specifically to fill this gap.

### The role of personality traits in end-of-life planning

Five key PTs, openness to experience, conscientiousness, extraversion, agreeableness, and neuroticism (John et al. 2008; John and Srivastava 1999; McCrae and Costa 2008) are identified by the Big Five factor model, which is a widely accepted and influential psychological framework for understanding and measuring personality. Rooted in broader temperamental tendencies, PTs reflect how individuals behave, process information, regulate emotions, and interpret life experiences (John et al. 2008; John and Srivastava 1999; McCrae and Costa 2008). These traits develop during childhood and mature adulthood, reflecting individual differences in thinking, feeling, and behaviour (McCrae and Costa 2008). Research shows PTs can slightly change across the lifespan (Graham et al. 2020), influenced by life events (Bleidorn et al. 2018), with genetic and environmental factors also playing a role (Bleidorn et al. 2014).

PTs shape decision-making styles (Flynn and Smith 2007), and strategies (e.g. Aarabi et al. 2022; Airaksinen et al. 2021; Marks and Lutgendorf 1999), influence healthcare preferences (Lattie et al. 2016), well-being (Mueller et al. 2019), and health literacy (Ryser et al. 2023) and have been directly linked to engagement in EoL planning (Ha and Pai 2012). Together, they reflect cognitive, emotional, and existential orientations, like autonomy, control, and preparedness, that are central to navigating complex EoL decisions. Taken together, the literature suggests that PTs reflect how individuals approach the multifaceted process of EoL planning.

More precisely, individuals higher on openness to experience tend to exhibit creativity and receptiveness to aesthetics, display imagination and intellectual curiosity, which collectively reflect a broader cognitive style characterized by exploration, flexibility, and a willingness to engage with complex or unfamiliar information (e.g. John et al. 2008; John and Srivastava 1999). These underlying facets, particularly tolerance for ambiguity and a propensity to seek out new knowledge, provide a clearer rationale for why openness may be relevant to EoL planning. Because EoL decisions often involve navigating uncertain medical scenarios, considering unfamiliar care options, and integrating complex information, individuals high in openness may be more inclined to actively seek information about care needs and available options (Sörensen et al. 2008; Ha and Pai 2012). Their cognitive curiosity and comfort with novel or uncertain situations may also translate into a preference for greater involvement in healthcare decisions, as they are more motivated to understand and evaluate different possibilities rather than avoid or defer such discussions (Flynn and Smith 2007). Conscientiousness characterizes individuals who tend to be goal-oriented, with a sense of responsibility, diligence, competence, self-discipline, and strong planning and organizational skills (e.g. John et al. 2008; John and Srivastava 1999). These facets suggest that conscientious individuals are generally motivated to anticipate future needs, reduce uncertainty, and maintain a sense of order and control in important life domains. It has been shown that this trait is important for EoL planning (Ha and Pai 2012). Individuals with higher levels of conscientiousness may be more proactive in seeking information and articulating their EoL wishes because their tendency towards preparation, responsibility, and long-term planning extend to healthcare decision-making (Flynn and Smith 2007). In addition, this proactive approach fosters a sense of control and autonomy, alleviating distress (Carr 2003; Detering et al. 2010). Planning and regulating one’s own EoL trajectory presents some benefits encompassing psychological, relational, and practical dimensions (Malhotra et al. 2022). Higher levels of extraversion characterize sociable individuals whose interests and energies are directed outward towards people and things rather than inward towards subjective experiences (e.g. John et al. 2008; John and Srivastava 1999). Agreeableness reflects higher cooperation, selflessness, and trust in others (e.g. John et al. 2008; John and Srivastava 1999). Because agreeable individuals tend to value harmonious relationships and rely on others in times of need, they may be more attentive to the implications of future health situations and more willing to engage in discussions about care preferences. This trait also seems to be related to a better awareness of future care needs (Sörensen et al. 2008), and this relational orientation may help explain why this trait is associated with a greater likelihood of formal EoL care planning (Ha and Pai 2012). Finally, neuroticism related to higher levels of negative emotions (anxiety, anger, insecurity, impulsiveness, etc.) and irritability (e.g. John et al. 2008; John and Srivastava 1999) might also play a role at the EoL because individuals high in neuroticism tend to avoid situations that trigger emotional discomfort or uncertainty, including discussions about future health decline. This trait seems related to the reluctance of any EoL planning forms (Lattie et al. 2016).

### The present study

Despite extensive research on the benefits of EoL planning (Carr and Luth 2016; Detering et al. 2010; Sudore et al. 2008) and the influence of PTs on a broad range of thinking, feeling, and health-related behaviours options (e.g. Aarabi et al. 2022; Airaksinen et al. 2021; Flynn and Smith 2007; Lattie et al. 2016; Marks and Lutgendorf 1999; Mueller et al. 2019; Ryser et al. 2023), there is a notable absence of nationally random studies that directly examine the relationship between PTs and EoL planning among older adults which can limit the generalizability of their findings. For instance, some research draws participants from private internal medicine practices and hospital-affiliated clinics (Sörensen et al. 2008). These settings may not reflect the broader population’s experiences and preferences. Other studies focus on specific cohorts like graduates from Wisconsin high schools (Flynn and Smith 2007; Moorman, 2011), which may not capture the general population’s diversity. Furthermore, some samples are composed of individuals with specific conditions, such as cancer patients (Lattie et al. 2016), leading to very specific and potentially non-representative insights. These limitations highlight the need for more inclusive and representative sampling methods to understand better EoL planning across diverse populations. These limitations hinder our understanding of EoL planning mechanisms and how personality influences it on a broader scale.

Our research aims to fill this gap by investigating the relationship between PTs and EoL planning using the Swiss SHARE data sample. This dataset provides a more comprehensive view of the population by providing a nationally random sample of adults, including older adults in various living situations.

Based on the theoretical framework presented above, we explore the following hypotheses. Openness to experience has been linked to active information-seeking regarding care needs and options (Sörensen et al. 2008), greater involvement in healthcare decision-making (Flynn and Smith 2007), and a higher likelihood of engaging in EoL discussions (Ha and Pai 2012). Accordingly, individuals high in openness might seek more flexible and exploratory discussions and may be more inclined to contemplate mortality and undertake preparatory actions such as expressing care preferences, addressing legal arrangements (e.g. wills, powers of attorney), appointing a healthcare proxy, and drafting a living will. Conscientiousness, similarly, is associated with proactive health-related behaviours and structured decision-making (Flynn and Smith, 2007; Ha and Pai 2012) and might be related to detailed and structured planning. We expect this trait to be particularly relevant for the organizational dimensions of EoL planning, including articulating wishes for the final stage of life and formalizing legal and medical directives. The relational aspect of EoL planning might be very important for individuals higher on extraversion. Extraversion has been linked to greater awareness of future care needs (Sörensen et al. 2008), which may translate into increased engagement with EoL considerations. Individuals scoring high on this trait may be more likely to communicate their preferences and complete relevant documentation. Agreeableness, through its association with interpersonal sensitivity and planning behaviours (Sörensen et al. 2008), may also foster engagement with the practical and relational aspects of EoL preparation, such as discussing care preferences and designating proxies. In contrast, neuroticism, characterized by emotional instability and heightened distress, has been associated with avoidance of EoL planning (Lattie et al. 2016). Individuals high in neuroticism may be less willing to engage with any of the components examined in this study, reflecting a tendency to evade emotionally charged decisions.

Overall, PTs might significantly impact how individuals experience and navigate their EoL journey, making them essential considerations in EoL planning. Understanding how PTs influence EoL planning can help healthcare providers tailor their approaches to meet individual needs more effectively. Understanding the barriers that prevent EoL planning might help design interventions that specifically address and mitigate these challenges.

If the relationship between PTs and EoL planning is better understood, more personalized, effective, and compassionate approaches to EoL care might be developed. Therefore, the EoL experiences and outcomes for individuals and their families might be improved.

## Methods

### Data, sample

This study uses a subsample of data from SHARE (Börsch-Supan 2022; Börsch-Supan et al. 2013). SHARE is a biennial longitudinal survey initiated in 2004 across 27 European nations and Israel. This interdisciplinary survey employs Computer-Assisted Personal Interviewing (CAPI) techniques, focusing on health, socioeconomic status, and social networks of individuals aged 50 and above. SHARE’s sampling strategy aims for national representation of individuals aged 50 and older. SHARE data are notable for internationally harmonized face-to-face interviews and optional country-specific paper-and-pencil questionnaires.

The present study utilizes data from both SHARE’s regular face-to-face interviews and a specialized, country-specific, paper-and-pencil self-administered questionnaire on EoL issues distributed in Switzerland during wave 6 (2015) after the regular interviews. The questions related to PTs were introduced in SHARE wave 7 (2017) and were asked for the first and only time during that wave. Like the regular SHARE sample, the Swiss SHARE sample is designed to be nationally representative of community-dwelling individuals aged 50 and older, along with their partners. This sample is periodically refreshed to maintain its target population. Since the Swiss SHARE sample was last refreshed in 2011, only adults aged 55 and over were included in our analyses. Respondents aged 50–54 in 2015 could only enter SHARE as partners and are, therefore, not representative of the general population aged 50–54. For further information on the eligibility rules, see the SHARE Release Guide 9.0.0 (SHARE-ERIC 2024).

**Fig. 1:**
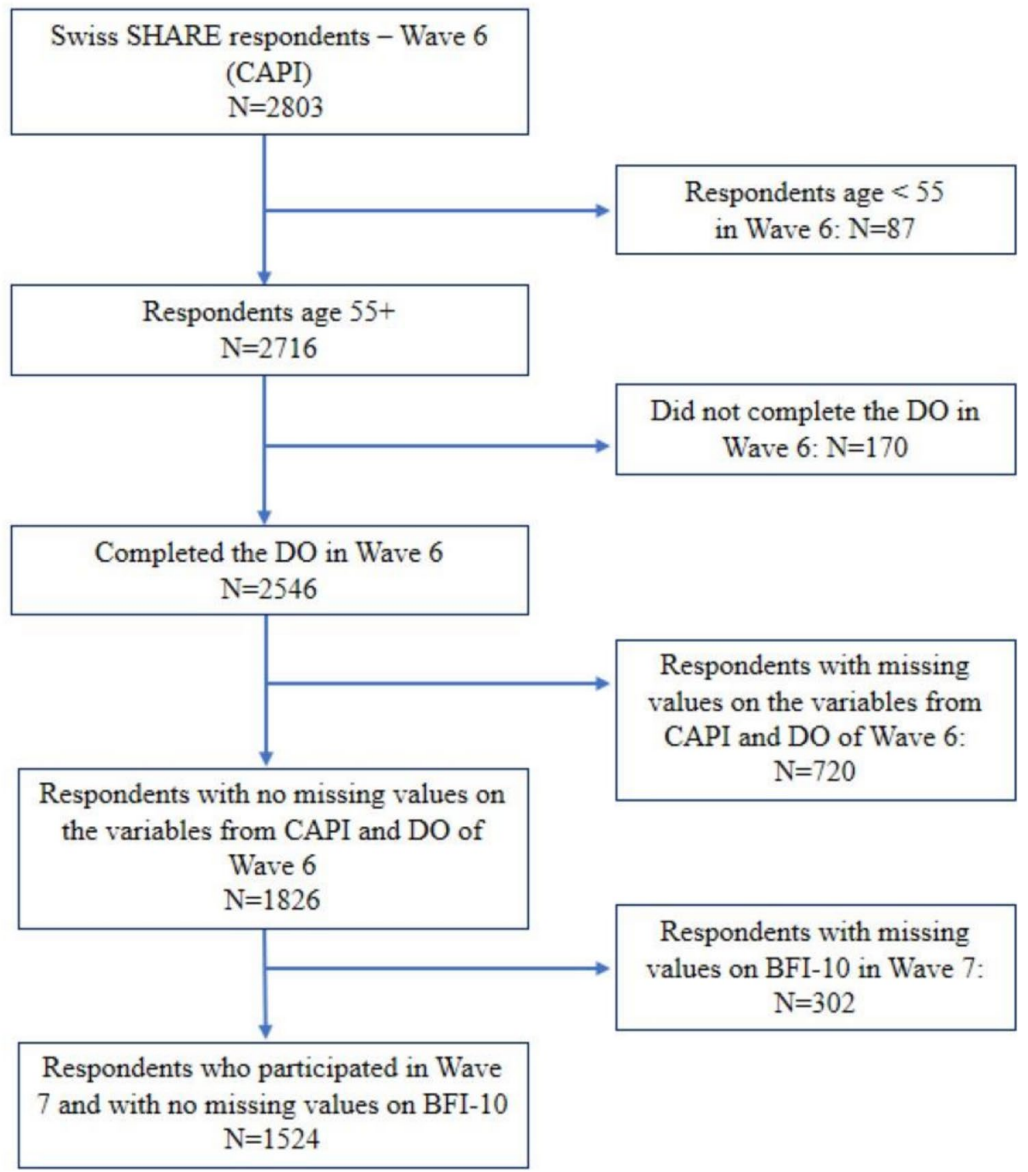
Study flow chart. *Source*: SHARE Authors own calculations. CAPI: computer-assisted personal interview, regular SHARE face-to-face interviews DO: Drop off, the country-specific paper-and-pencil self-administered questionnaire about end-of-life issues distributed in Switzerland in waves 6 (2015) BFI-10 is the Big Five Inventory – BFI-10

The analytical sample comprises individuals aged 55 and older who participated in the harmonized SHARE face-to-face interviews during waves 6 and 7, and who also completed the Swiss paper-and-pencil self-administered EoL questionnaire in wave 6. Following this selection process, the final analytical sample includes 1,524 respondents. A detailed overview of missing data types and exclusion criteria is provided in Fig. 1, Appendix 1, and Appendix 2, demonstrating that item non-response within conducted interviews (including incomplete modules and missing item blocks) is the primary driver of case loss, whereas pure unit non-response is comparatively low.

### Dependent variables

Our study examined seven specific and independent dimensions of thoughts and actions of EoL planning aspects distributed across different sections of the questionnaire: two categorical questions (with four response categories), which are: 1. avoiding thinking about death; 2. thinking about wishes for the last months of life; and five binary questions (no/yes) which are: 3. having discussed EoL preferences; 4. having a testament; 5. having a power of attorney; 6. having designated a healthcare proxy; and 7. having a living will. These items were developed based on Carr’s seminal work (Carr 2003, 2012; Carr and Luth 2016, 2017, 2019) and adapted to the Swiss legal context. For the analyses, the two categorical questions were recoded (dichotomized) to have coding identical to the binary questions. This recoding allows us to directly compare the regression coefficients that describe the relationship between PTs and the stages of EoL planning.

Each aspect was measured using a single question*. Avoiding thinking about death* was measured by requesting participants to indicate their level of agreement with the following statement: “I avoid thinking about death as much as possible”. The responses ranged on a four-point Likert scale from “strongly agree”, “agree”, “disagree”, to “strongly disagree”. For analysis, responses were dichotomized, with “strongly agree” and “agree” coded as 0, whereas “disagree” and “strongly disagree” coded as 1. *Thinking about wishes for the last months of life* was measured by asking participants the following question: “Prior to today, how often have you thought about your wishes for the last months of your life?”. The response options included “never”, “rarely”, “sometimes”, and “often”. For analysis, responses were dichotomized, with “rarely” and “never” coded as 0, and “sometimes” and “often” coded as 1. For the five remaining aspects—discussing EoL preferences, having a testament, having a power of attorney, designating a healthcare proxy, and having a living will—participants responded with “no” coded as 0 or “yes” coded as 1. *Having discussed EoL preferences* is based on whether participants had ever discussed their EoL preferences with someone. For *Having a testament*, participants were asked whether they had a testament that detailed what should happen to their possessions in case of death. *Having a power of attorney* is evaluated by asking participants whether they had designated a power of attorney for financial, legal, or administrative matters if they lost their decision capacity. For *Having designated a healthcare proxy*, participants who indicated that they had a person of trust to make medical decisions on their behalf were also asked whether they had appointed someone in writing to make medical decisions if they lost their decision-making capacity. *Having a living will* indicates whether participants had completed a written statement outlining their wishes and refusals for medical treatments and care.

To avoid misunderstandings, we provided respondents with clear definitions of less familiar procedures, like living wills and healthcare proxy appointment, so they could fully grasp the concepts, strengthening the reliability and validity of our measures. Appendix 3 presents the Phi correlations between EoL planning variables.

### Independent variables

PTs were assessed using the shortened 10-item version of the Big Five Inventory—BFI-10 (Rammstedt and John 2007). The BFI-10 includes two statements for each of the five personality factors: openness to experience, conscientiousness, extraversion, agreeableness, and neuroticism. Participants rated the statements aimed at measuring each PTs on a 5-point scale, with response options ranging from “strongly disagree” (1) to “strongly agree” (5). Since each PTs is assessed based on two items, the corresponding trait scores are calculated as the means of the two items referring to a given trait. As a result, the measures for each PTs can take on values ranging from 1 to 5 with an increment of 0.5 unit. Appendix 4 presents the correlations computed between the two items representing each Big Five dimension. In addition, the validity of the measure is thoroughly addressed and developed in the methodological documentation of the SHARE survey (Bergmann et al. 2019).

### Control variables

Previous research has demonstrated important associations of sociodemographic variables with selected EoL planning outcomes in Switzerland (e.g. Vilpert et al. 2018). We adjust our regression models for sociodemographic characteristics to avoid potential confounding issues.

Our control variables are gender (0 = male, 1 = female), age groups (55–64 years; 65–74 years; 75 + years), and level of education according to the International Standard Classification of Education (ISCED) 2017 (Hoffmeyer-Zlotnik and Wolf 2003) where primary education group comprises ISCED levels 0–1–2, secondary education includes ISCED levels 3–4, and tertiary education contains levels ISCED 5–6. We also include the partnership status, i.e. whether the respondents live with a partner (0 = has a partner, 1 = has no partner), and the parenthood status (having or not living children). We use a variable that assesses respondents’ subjective financial situation based on the household’s self-rated difficulty in making ends meet. The three permissible answer categories for this variable were making ends meet “easily”, “fairly easily”, or “with difficulty”. We include the respondents’ living environment (urban or rural) and the three Swiss linguistic regions: German-speaking, French-speaking, and Italian-speaking. Finally, we selected two variables assessing the respondents’ health status: self-rated health status, whose response categories have been combined into excellent/(very) good health and fair/poor health; self-reported limitations in activities of daily living (ADL) (Katz et al. 1970) recoded into a binary variable, i.e. “no ADL limitations” versus “one or more ADL limitations”.

### Analytical strategy

All analyses are conducted using unweighted data. Following current practice in longitudinal studies of older adults, where complete-case analysis remains the predominant and operationally robust approach (Okpara et al., 2022), we conducted complete-case analyses. This approach provides transparency and avoids additional assumptions required by imputation-based methods.

We described the main sociodemographic traits of our sample and the seven EoL planning components using proportions. For respondents’ PTs, we reported the average values and standard deviations.

We then used seven separate logistic regressions to assess the partial associations of the seven independent components of EoL planning with respondents’ PTs, adjusting for potential confounders. The multivariable models controlled for key sociodemographic and health-related characteristics, including gender, age group, educational attainment, partnership status, parenthood, subjective financial situation, living environment, linguistic region within Switzerland, self-rated health, and limitations in activities of daily living. This adjustment ensures that the estimated associations between PTs and EoL planning components are not confounded by these covariates.

Model estimates are presented as average marginal effects (AMEs), expressed in percentage points. AMEs quantify the change in the average conditional probability of the outcome (P(Y = 1|X)) when the explanatory variable shifts from 0 to 0.5, holding all other covariates at their observed values. This metric facilitates intuitive interpretation of effect sizes across models.

To account for potential intra-household dependencies, particularly among partnered respondents, standard errors were clustered at the household level. All analyses were performed using STATA/SE 17.0 (StataCorp LLC, College Station, TX). Replication codes are available from the authors upon request.

## Results

Table 1 (unweighted data) provides a comprehensive overview of the estimated sociodemographic characteristics of our analytical sample, the mean PTs scores in 2017, and the distribution of the seven EoL planning components measured in 2015. These descriptive findings underscore the sample’s varying engagement in EoL planning.

**Table 1:** Sample characteristics, adults aged 55 + , SHARE Switzerland 2015 and 2017, n = 1524; unweighted data

	Proportions (%)
Social characteristics	
Gender	
Male	47.6
Female	52.4
Age groups	
55–64 years	39.2
65–74 years	37.3
75 + years	23.5
Education	
Basic	17.5
Secondary	64.3
Tertiary	18.2
Living with a partner	
Yes	78.1
No	21.9
Parenthood	
No children	15.6
At least on child	84.4
Make ends meet	
Easily	58.9
Fairly easily	29.5
With difficulty	11.7
Regional characteristics	
Living environment	
Urban	53.8
Rural	46.2
Switzerland linguistic regions	
German	74.3
French	22.9
Italian	2.8
Health characteristics	
Self-rated health	
Excellent/(Very) good health	85.1
Poor/fair health	14.9
Self-reported limitations in activities of daily living (ADLs)	
No limitation	95.5
At least one limitation	4.5
EoL planning dispositions	
Avoiding thinking about death	
(Strongly) agree	42.3
(Strongly) disagree	57.7
Thinking about wishes for last months of life	
Rarely/Never	62.9
Often/Sometimes	37.1
Having discussed EoL preferences	
No	49.0
Yes	51.0
Having a testament	
No	62.6
Yes	37.4
Having a power of attorney	
No	61.4
Yes	38.6
Having designated a healthcare proxy	
No	84.0
Yes	16.0
Having a living will	
No	78.8
Yes	21.2
Personality traits	
Openness	3.7 (0.9)
Conscientiousness	4.3 (0.7)
Extraversion	3.5 (1)
Agreeableness	3.7 (0.8)
Neuroticism	2.5 (1)

Source SHARE Authors’ own calculations. Personality traits’ statistics are mean (standard deviation).

The average age of the sample is 67 years (standard deviation 8.7). Regarding PTs, participants scored an average of 3.7 in openness to experience, 4.3 in conscientiousness, 3.5 in extraversion, 3.7 in agreeableness, and 2.5 in neuroticism. Regarding EoL planning attitudes and behaviours, 42.3% of participants indicated that they (strongly) agree with avoiding thinking about death, while 57.7% (strongly) disagree. When reflecting on their wishes for the last months of life, 62.9% of respondents reported rarely or never engaged in such thoughts, whereas 37.1% did so at least sometimes. About half of respondents (51.0%) stated that they had discussed their EoL preferences with someone. Regarding more formal EoL planning behaviours, 37.4% of participants reported having a testament, and 38.6% reported having a power of attorney. Regarding EoL care planning, 16.0% of participants indicated they had designated a healthcare proxy, while 21.2% reported having a living will.

Table 2 (unweighted data), shows the results from our multivariable logistic regression models of EoL planning components on respondents’ PTs, controlling for social, regional, and health characteristics. Concerning the sociodemographic variables, our results demonstrate that gender, age, education, living situation, parenthood, financial status, living environment, linguistic region, self-rated health, and limitations in activities of daily living all play significant roles in various aspects of EoL planning. Females, older adults, and those with higher education levels are generally more proactive in EoL planning. Conversely, individuals facing financial difficulties or residing in French-speaking regions are less likely to engage in such planning. Our results align with previous research highlighting the relationship between sociodemographic variables and aspects of advance directives (e.g. Vilpert et al. 2018).

**Table 2:** Average marginal effects (AMEs) based on logistic regressions of EoL planning dispositions on personality traits, controlling for social,  regional, and health characteristics, adults aged 55 + , SHARE Switzerland, 2015/2017, n = 1524. Unweighted data

	Avoiding thinking about death (strongly) disagree)	Thinking about wishes for last months of life (often/sometimes)	Having discussed EoL preferences (yes)	Having a testament (yes)	Having a power of attorney (yes)	Having designated a healthcare proxy (yes)	Having a living will (yes)
Personality traits							
Openness	4.9^***^	3.5^*^	3.1^*^	3.0^*^	3.6^**^	1.1	0.8
Conscientiousness	−2.1	−0.9	1.2	2.4	2.9	1.6	1.0
Extraversion	0.3	0.8	0.8	3.8^**^	1.2	1.8	0.0
Agreeableness	1.3	1.6	0.9	−1.3	−2.4	−1.3	0.5
Neuroticism	1.0	4.0^**^	1.3	1.4	1.1	0.6	−0.5
Social, regional, and health characteristics							
Gender (ref: male)							
Female	12.2^***^	12.5^***^	15.9^***^	−1.3	3.6	4.9^**^	9.4^***^
Age groups (ref: 55–64)							
65–74	−0.1	2.6	2.1	13.4^***^	8.6^**^	7.2^***^	8.5^***^
75 +	−7.7^*^	5.3	2.8	24.2^***^	23.6^***^	14.6^***^	18.8^***^
Education (ref: basic)							
Medium	8.4^*^	−4.1	7.5^*^	4.3	−1.5	0.9	0.9
High	18.0^***^	2.1	8.7	7.2	−2.1	5.5	5.0
Living with a partner (ref: yes)							
No partner	0.0	-6.0	8.1^*^	10.4^***^	−8.6^**^	−2.3	−2.5
Parenthood (ref: no children)							
Any children	−7.2^*^	−3.9	2.5	−26.7^***^	−6.3	−5.8	−5.6
Make ends meet (ref: easy)							
Fairly easily	−3.9	1.1	−2.9	−4.5	−5.7	−1.1	−0.5
With difficulty	−11.2^**^	−5.2	−1.0	−15.9^***^	−9.0^*^	−2.3	−4.9
Living environment (ref: urban)							
Urban area	1.7	1.7	2.8	6.5^*^	5.0	−1.7	0.9
Switzerland linguistic regions (ref: German)							
French	−19.5^***^	−19.9^***^	−16.5^***^	−4.5	−20.9^***^	−11.7^***^	−18.1^***^
Italian	−3.1	−8.6	−15.6	−14.3^*^	−14.1^*^	−3.9	−11.8
Self-rated health (ref: Excellent/(very) good)							
Fair/poor	−2.0	3.2	−5.1	−5.8	−3.5	−5.2^*^	−1.8
Self-reported limitations in activities of daily living (ADLs) (ref: no limitation)							
1 + ADL limitations	5.5	3.4	14.8^*^	3.3	8.1	7.0	7.0
N	1524	1524	1524	1524	1524	1524	1524

Source SHARE Authors ‘own calculations. Average marginal effects (AMEs) based on logit regression models. All probabilities are multiplied by 100 to express AMEs in percentage points. Asterisks indicate significance levels: *** < 0.1%, ** 1%, *5%. Interpretation of AMEs: Each unit of openness to experience increases the probability of (strongly) disagreeing with the statement “I avoid thinking about death” by 4.9 percentage points.

Concerning the PTs, higher level of openness was statistically significantly positively associated with more engagement in EoL planning. Specifically, for each unit increase in openness to experience among participants, the probability of (strongly) disagreeing with the statement “I avoid thinking about death” rose by 4.9 percentage points (AME: 4.9; *p* < 0.001). Similarly, respondents with higher levels of openness to experience were more likely to think about their wishes for their last months of life (AME: 3.5; *p* < 0.05). Higher openness to experience was also associated with discussing one’s EoL preferences (AME: 3.1; *p* < 0.05). Finally, higher levels of openness to experience were also positively related to having a testament (AME: 3.0; *p* < 0.05) and having a power of attorney (AME: 3.6; *p* < 0.01). Two other PTs were statistically significantly positively associated with two EoL planning attitudes and behaviours. Higher levels of extraversion were positively related to having a testament (AME: 3.8; *p* < 0.01), while higher levels of neuroticism were positively associated with thinking about wishes for the last months of life often or sometimes (AME: 4.0; *p* < 0.01).

## Discussion

To the best of our knowledge, this study is the first to examine the contribution of PTs to multiple components of EoL planning from a holistic perspective, using a nationally random sample. Our findings show how PTs relate to engagement in EoL planning and whether they may act as barriers. Since EoL planning is strongly linked to better quality of life in later life (Carr and Luth 2016, 2017), encouraging such planning should remain a key policy priority. Using information on PTs could also help make outreach efforts more efficient and better tailored to individuals.

The study reveals nuanced associations between specific PTs—openness to experience, extraversion, and neuroticism—and distinct components of EoL planning, controlling for the influence of social, regional, and health-related factors. Individuals with higher levels of openness displayed consistently more favorable attitudes and behaviours towards EoL planning. These individuals more commonly disagreed with the idea of avoiding thoughts about death, were more inclined to reflect on their EoL preferences, and indicated a willingness to engage in this generally challenging topic actively. Our results align with prior research: Openness is linked to awareness of future care needs, seeking information about care options (Sörensen et al. 2008), and frequent reminiscence addressing life’s meaning, reducing fear of death, and confronting mortality (Cappeliez and O’Rourke 2002).

Our findings suggest that individuals higher on openness are more receptive to discussing sensitive topics like death, often considered taboo in Western societies (Wise 2012), due to their readiness to embrace new perspectives (e.g. McCrae and Costa 2008), even if it means deviating from conventional norms. However, our analysis indicates no significant predisposition among individuals expressing higher levels of openness to create a living will or designate a healthcare proxy compared to others. It is worth noting that at the time of our 2015 survey, these tools had only been introduced into the Swiss Civil Code for two years (2013). Many, including those high in openness, may have been unaware of them. Future research should explore whether individuals with higher levels of openness are more engaged in EoL care planning once these measures are well-established and widely known.

A second PTs related to some EoL attitudes or behaviours is extraversion. Extraversion is significantly associated with having a testament, possibly due to increased social engagement for individuals expressing higher levels of extraversion (e.g. McCrae and Costa 2008). We assume that having a testament is viewed as socially responsible, especially for those with extensive social ties who wish to ensure precise asset distribution after death. Higher levels of extraversion tend to be linked with engaging more in EoL planning than lower levels of extraversion.

Finally, the results demonstrated that individuals with higher scores on neuroticism express frequent thoughts about wishes for the last months of life. Those individuals may more strongly perceive the emotional aspects of the EoL, focusing on their anxieties and fears rather than engaging in formal legal and healthcare arrangements for their death. As a result, individuals with higher levels of neuroticism may refrain from documenting their EoL preferences in legal instruments. This result is consistent with existing literature indicating that individuals with higher levels of neuroticism are less skilled in problem-solving and proactive coping (Flynn and Smith 2007; Sörensen et al. 2008), making them less likely to formalize their EoL preferences in documents like testaments and living wills.

In summary, these findings tend to indicate that PTs linked to some aspects of EoL planning are associated with three key factors. First is the ability to think and act outside normative frameworks and pursue less conventional avenues when they perceive them as pertinent. Second, the individual’s capacity to take proactive steps when confronting challenging topics, such as death. Third, the capacity of individuals to tolerate ambiguity, remain open to internal states, and explore negative emotions. Proactive PTs, such as extraversion and openness to experience, may motivate them to engage in various aspects of EoL planning. Surprisingly, conscientiousness is a PTs that does not play a role in EoL planning despite its link with organization and planning (e.g. McCrae and Costa 2008).

### Limitations

This study offers useful insights into links between PTs and EoL planning, but several limitations remain. First, although the data stem from a population-based survey with a high response rate, potential selection bias remains due to non-response and attrition, common challenges in longitudinal studies such as SHARE (e.g. Banks et al. 2011; Gaertner et al. 2016; Tinker et al. 2009). Missing data in this context often reflect meaningful decline and disproportionately affect vulnerable groups, including women, individuals aged 75 and older, those with lower educational attainment, and respondents with health limitations (additional analyses available from the authors comparing the unit versus item non-response sample with the complete cases sample). As the analyses relied on complete cases, one limitation of our findings is that they are most generalizable to older adults who are relatively healthy and able to participate fully in survey research. While this approach excludes more vulnerable individuals, we assume that it also provides a clearer picture of patterns and associations within a cognitively and physically robust segment of the ageing population.

Second, ultra-short personality measures such as the BFI-ten save time by avoiding item redundancy but may not match the depth of regular assessments. A limitation in the present study is that the correlations between BFI-ten items were moderate to low. We nevertheless retained the trait scores because each provides distinct and theoretically meaningful information about its dimension. Future research may explore narrower traits like painfulness and tolerance for ambiguity in EoL contexts and consider an additional honesty-humility factor (Ashton et al. 2014).

Third, our decision to dichotomize the two four-category ordinal items presents both advantages and limitations. Dichotomization enhances parsimony and interpretability, making results easier to communicate and compare across outcomes. However, this approach also entails methodological drawbacks: it reduces variability and statistical power. It may mask more nuanced associations that could have been captured through analyses preserving the full ordinal structure.

Finally, our findings align with trends in WEIRD (Western, Educated, Industrialized, Rich, and Democratic) countries, suggesting that certain EoL planning behaviours and the role of PTs may be broadly relevant. However, variations in cultural attitudes, legal frameworks, and healthcare systems (Searight and Gafford 2005) can limit international generalizability. These contextual factors must be considered when applying our results across settings.

## Conclusions

Our findings suggest that PTs play a limited role in shaping attitudes towards EoL planning, indicating that no specific PTs poses a major barrier to its broader adoption. Individuals across personality profiles appear receptive to such planning, though tailored communication strategies may improve engagement. Our findings highlight the need for a message that normalizes EoL discussions. Ultimately, policy efforts should aim to destigmatize this life phase and make it accessible to all, regardless of personality.

## Data Availability

This article uses data from Börsch-Supan, A. (2022). Survey of Health, Ageing and Retirement in Europe (SHARE) Wave 8. Release version: 8.0.0. SHARE-ERIC. Data set. 10.6103/SHARE.w8.100. Study data that have already been deidentified are available to the scientific community upon submission of a data requestion application to the SHARE study.

## Appendix 1

See Table 3.

**Table 3:** Perspectives from wave 7 Interviews: understanding attrition, item non-response, and unit non-response

Interview done in wave 7										
	No interview		Main interview		Mortality follow-up		Missing		Total	
Dropped cases	N	%	N	%	N	%	N	%	N	%
Final analytical sample	0	0	1524	100	0	0	0	0	1524	100
Respondent age < 55 W6	8	9,2	65	74,7	0	0	14	16,1	87	100
Did not complete DO in W6	4	2,4	113	66,5	10	5,9	43	25,3	170	100
Missing values in CAPI or DO W6	16	2,2	564	78,3	22	3,1	118	16,4	720	100
Missing values on Big Five Inventory	45	14,9	17	5,6	28	9,3	212	70,2	302	100
Total	73	2,6	2283	81,4	60	2,1	387	13,8	2803	100

SHARE Authors ‘own calculations. CAPI: computer-assisted personal interview, regular SHARE face-to-face interviews. DO: Drop off the country-specific paper-and-pencil self-administered questionnaire about EoL issues distributed in Switzerland in waves 6 (2015). BFI-10 is the Big Five Inventory. Mortality follow-up: Mortality questionnaire for individuals who died in Waves 6 or 7 and had participated in Wave 6.

Additional analyses (available from the authors) demonstrated that the final analytical sample is more likely to be partnered, healthier, and with fewer functional limitations, and somewhat less represented among the oldest old.

## Appendix 2

See Table 4.

**Table 4:** Source of the variables selected for the study and proportion of missing responses by item, adults aged 55 + , SHARE Switzerland 2015 and 2017, n = 2546

	Wave	Questionnaire	Missing response (%)
Gender	6	CAPI	0
Age groups	6	CAPI	0
Education	6	CAPI	2
Living with a partner	6	CAPI	0.4
Parenthood	6	CAPI	0.1
Make ends meet	6	CAPI	2.3
Living environment	6	CAPI	4.2
Switzerland linguistic regions	6	CAPI	0
Self-rated health			0.1
Self-reported limitations in activities of daily living (ADLs)	6	CAPI	6.2
Avoiding thinking about death	6	DO	3.1
Thinking about wishes for last months of life	6	DO	2.5
Having discussed EoL preferences	6	DO	3.6
Having a testament	6	DO	6.2
Having a power of attorney	6	DO	8.9
Having designated a healthcare proxy	6	DO	9.7
Having a living will	6	DO	4.8
Personality traits^a^			
Openness to experience	7	CAPI	18
Conscientiousness	7	CAPI	18
Extraversion	7	CAPI	18
Agreeableness	7	CAPI	18
Neuroticism	7	CAPI	18

Source SHARE Authors ‘own calculations.

N = 2546 is based on respondents aged 55 + who participated in CAPI wave 6 and completed the Swiss DO wave 6. CAPI: computer-assisted personal interview, regular SHARE face-to-face interviews. DO: Drop off, the country-specific paper-and-pencil self-administered questionnaire about EoL issues distributed in Switzerland in waves 6 (2015). ^a^Respondents present in Wave 6 (2015), but who did not participate in Wave 7 (2017).

## Appendix 3

See Table 5.

**Table 5:** Phi correlations between EoL planning variables

Variables	Avoiding thinking about death	Thinking about wishes for last months of life	Having discussed EoL preferences (yes)	Having a testament	Having a power of attorney	Having designated a healthcare proxy	Having a living will
Avoiding thinking about death (disagree)	–	0.237***	0.252***	0.053*	0.055*	0.055*	0.113***
Thinking about wishes for last months of life		–	0.315***	0.092***	0.197***	0.198***	0.220***
Having discussed EoL preferences (yes)			–	0.172***	0.277***	0.271***	0.325***
Having a testament (yes)				–	0.328***	0.265***	0.269***
Having a power of attorney (yes)					–	0.355***	0.340***
Having designated a healthcare proxy (yes)						–	0.684***

Source SHARE Authors ‘own calculations. All variables are binary (0 = no, 1 = yes). Reported values correspond to phi (φ) coefficients, which are mathematically equivalent to Pearson correlations when computed on dichotomous 0–1 variables. *p* < 0.05 = *, *p* < 0.001 = ***. N = 1524 for all correlations.

Overall, all variables were positively associated, indicating that individuals who engaged in one form of EoL (EOL) planning were generally more likely to engage in others. Intercorrelations among the seven binary outcomes were mostly small to moderate (*φ* ≈ 0.05–0.35).

## Appendix 4

See Table 6.

**Table 6:** Correlations between big five items

Variable	Active imagination	Thorough job	Outgoing, sociable	Finds fault with others (R)	Nervous easily
Few artistic interests (R)	0.248***				
Tends to be lazy (R)		0.213***			
Reserved (R)			0.423***		
Generally trusting				0.106***	
Relaxed, handles stress well (R)					0.297***

Note: Source SHARE Authors ‘own calculations. Items marked R are reverse-scored. Reverse-scored items: Few artistic interests (R) = "I see myself as someone who has few artistic interests"; Tends to be lazy (R) = "I see myself as someone who tends to be lazy"; Reserved (R) = "I see myself as someone who is reserved"; Finds fault with others (R) = "I see myself as someone who tends to find fault with others"; Relaxed, handles stress well (R) = "I see myself as someone who is relaxed, handles stress well". N = 1524 for all correlations. p < 0.001 = ***.
